# Client Perspectives of Case Stories in Internet-Delivered Cognitive Behavioral Therapy for Public Safety Personnel: Mixed Methods Study

**DOI:** 10.2196/64454

**Published:** 2024-10-25

**Authors:** Jill AB Price, Julia Gregory, Hugh C McCall, Caeleigh A Landry, Janine D Beahm, Heather D Hadjistavropoulos

**Affiliations:** 1 Canadian Institute for Public Safety Research and Treatment University of Regina Regina, SK Canada; 2 PSPNET University of Regina Regina, SK Canada; 3 Department of Psychology University of Regina Regina, SK Canada

**Keywords:** internet-delivered cognitive behavioral therapy, case stories, public safety personnel, public safety, mental health, internet interventions, digital mental health interventions, first responders

## Abstract

**Background:**

Internet-delivered cognitive behavioral therapy (ICBT) is an effective and convenient means of offering cognitive behavioral therapy to the general population. To increase access to ICBT among Canadian public safety personnel (PSP)—a group that experiences elevated rates of mental health concerns and barriers to mental health care—a clinical research unit called PSPNET has tailored ICBT to PSP, primarily through offering case stories and PSP-specific examples within an ICBT program. PSPNET’s first and most frequently used ICBT program, called the PSP Wellbeing Course, has been found to reduce symptoms of mental disorders (eg, anxiety, depression, and posttraumatic stress) among PSP. Little research, however, has investigated clients’ perceptions of the case stories in this course.

**Objective:**

This study was designed to expand the literature on the use and evaluation of case stories in ICBT among PSP. Specifically, this study investigated (1) PSP’s perceptions of the case stories using the theoretical model provided by Shaffer and Zikmund-Fisher and (2) PSP feedback on the case stories in the PSP Wellbeing Course.

**Methods:**

This study included 41 clients who completed the PSP Wellbeing Course. Of these, 27 clients completed a bespoke questionnaire called the Stories Questionnaire, 10 of whom also participated in a semistructured interview.

**Results:**

Findings show that perceptions of the case stories in the PSP Wellbeing Course were largely positive and that the case stories were generally successful in achieving the 5 purposes of case stories (ie, informing, comforting, modeling, engaging, and persuading) proposed by Shaffer and Zikmund-Fisher. Client feedback also identified 3 tangible areas for story improvement: characters, content, and delivery. Each area highlights the need for and potential benefits of story development. Not all PSP engaged with the case stories, though, so results must be interpreted with caution.

**Conclusions:**

Overall, this study adds to the growing body of research supporting the use of case stories in internet-delivered interventions among PSP.

**Trial Registration:**

ClinicalTrials.gov NCT04127032; https://www.clinicaltrials.gov/ct2/show/NCT04127032

## Introduction

### Background

The term “public safety personnel” (PSP) refers to individuals whose work ensures the safety and security of citizens (eg, border security officers, correctional workers, firefighters, Indigenous emergency managers, operational intelligence personnel, paramedics, police, public safety communicators, and search and rescue personnel [1]). Due to high-stress work environments and exposures to potentially psychologically traumatic events (PPTE), PSP are at high risk of mental health challenges [2,3], with 44.5% screening positive for at least 1 mental disorder [2]. PSP also often experience barriers to receiving treatment such as workplace stigma toward mental health, the desire to self-manage symptoms of mental disorders, limited time available to attend treatment, limited accessibility of mental health providers in rural areas, and treatment costs [4,5].

Internet-delivered cognitive behavioral therapy (ICBT) is an effective psychological treatment for various mental disorders that includes evidence-based cognitive behavioral therapy (CBT) techniques in a digital format [6]. An advantage of ICBT is that clients can access it privately at a time and place that is most convenient for them. ICBT can be disorder specific [6,7] or transdiagnostic (ie, designed to treat symptoms of several disorders) and can be delivered in a self- or therapist-guided format (eg, phone and email [8]). Clients are more likely to choose transdiagnostic over disorder-specific ICBT despite comparable treatment satisfaction and outcomes [9,10]. Overall, ICBT is similarly effective to face-to-face CBT in managing mental health challenges such as anxiety, depression, and posttraumatic stress [6].

A clinical research unit called PSPNET was launched in 2019 with funding from the Government of Canada to develop, implement, and evaluate ICBT tailored to the needs of Canadian PSP [11]. PSPNET adapted a previously established ICBT course that has excellent treatment outcomes in Australia [10] and Canada [12,13] to address common mental health concerns of PSP. The course was titled the PSP Wellbeing Course and, like the established ICBT course upon which it was based, is therapist guided and transdiagnostic. Mental health outcomes of the PSP Wellbeing Course demonstrate moderate to large, statistically significant declines in PSP’s anxiety, depression, and posttraumatic stress symptoms from before to after treatment [14,15]. The PSP Wellbeing Course was tailored via interviews and focus groups to better understand PSP’s preferences in ICBT [16] and to develop fictional client narratives called case stories. Of note, PSP Wellbeing Course findings [17] parallel other ICBT program findings, with many clients reporting the case stories as helpful but needing improvement [17-20].

Case stories are illustrative examples that are commonly used in health care services to provide anecdotes, analogies, and metaphors to facilitate learning and enhance the client experience. Case stories can be sourced from real or fictitious user profiles [21]. Fictitious user profiles, also known as personas, are designed to represent current or prospective clients from specific groups with detailed characteristics (eg, name, photo, symptoms, and diagnosis). Information used to develop such personas can be sourced from clients [22] or experts (eg, clinician experiences working with PSP [23]). Shaffer and Zikmund-Fisher [21] identified five main purposes of case stories in health care: (1) to inform clients of basic knowledge and lived experiences, (2) to provide comfort to clients by normalizing their experiences, (3) to model targeted behaviors, (4) to persuade clients to use targeted behaviors, and (5) to enhance client engagement. This taxonomy offers a valuable guideline for designing, implementing, and evaluating case stories [21].

Client perceptions of case stories in health care services [18,24,25] and in ICBT programs are generally positive [17-20], but clients often suggest stories could be improved. Preliminary findings show that PSP are also generally satisfied with case stories in ICBT, citing them as a valuable component that portrays authentic and relatable experiences [26] while providing some feedback to improve content and delivery. Content feedback from PSP has centered on improving story authenticity [16,17,26] and relatability [17]. For example, some clients request the use of real, rather than fictitious, case stories [16]. Other feedback includes increasing the complexity of stories [17,26] and adding more examples on the application of treatment principles [27]. Delivery feedback has centered on improving story accessibility (eg, adding audio or video narratives [17,26]).

The extant research provides preliminary support for the inclusion of case stories in ICBT tailored for PSP, but some gaps in knowledge still exist, highlighting the need for further story evaluation. Improving health care services often relies on client perceptions and feedback [25,28]. Client perceptions can identify treatment satisfaction [29] and sustain good practices (eg, reinforce good practices among clinicians [25]), while client feedback can be used to identify problems, inform areas for improvement, evaluate improvements, and assess progress toward organizational goals [28]. To date, story feedback in ICBT has largely been gained through open-ended, web-based questions asking clients to provide brief written feedback about what they liked and disliked about ICBT generally [18-20,26,27]. In-depth client perceptions of and feedback on case stories in ICBT among PSP have not yet been obtained.

### Research Questions and Hypotheses

This study was designed to expand the literature on the use and evaluation of case stories in ICBT among PSP to help identify what is effective and ineffective about case stories and how limitations can be addressed. Specifically, this study investigated (1) PSP’s perceptions of the case stories using the theoretical model provided by Shaffer and Zikmund-Fisher [21] and (2) PSP’s feedback on the case stories in the PSP Wellbeing Course. The research team hypothesized that (1) PSP’s perceptions of the case stories would be generally positive, (2) PSP’s perceptions would align with the purposes of stories identified by Shaffer and Zikmund-Fisher [21], and (3) PSP would provide tangible feedback on ways to improve case stories in the PSP Wellbeing Course.

## Methods

### Ethical Considerations

This mixed methods study was approved by the Research Ethics Board at the University of Regina (#2019-157) and was carried out within the context of a longitudinal, single-group, open trial registered on ClinicalTrials.gov (NCT04127032). Clients provided informed consent after being made aware of the study details and the potential benefits and risks of participation. Participants were provided with access to the PSP Wellbeing Course but were not offered other incentives to encourage participation. Client data were stored on a secure server and were deidentified prior to analyses.

### Study Design

Prospective clients signed up for a PSPNET account; provided informed consent to participate; and completed eligibility screening questionnaires that collected demographic, clinical, and occupational information. This was followed by a telephone interview, after which eligible clients were enrolled in an ICBT course appropriate to their symptoms and preferences. This study describes outcomes only for clients enrolled in the therapist-guided PSP Wellbeing Course (English version). At 6 weeks after enrollment, clients were invited to schedule a semistructured interview for around the 8-week postenrollment time point. At 8 weeks after enrollment, we administered the Stories Questionnaire, a bespoke questionnaire designed to assess perceptions of and feedback on the case stories (described in detail below).

### Participants

**Figure 1:**
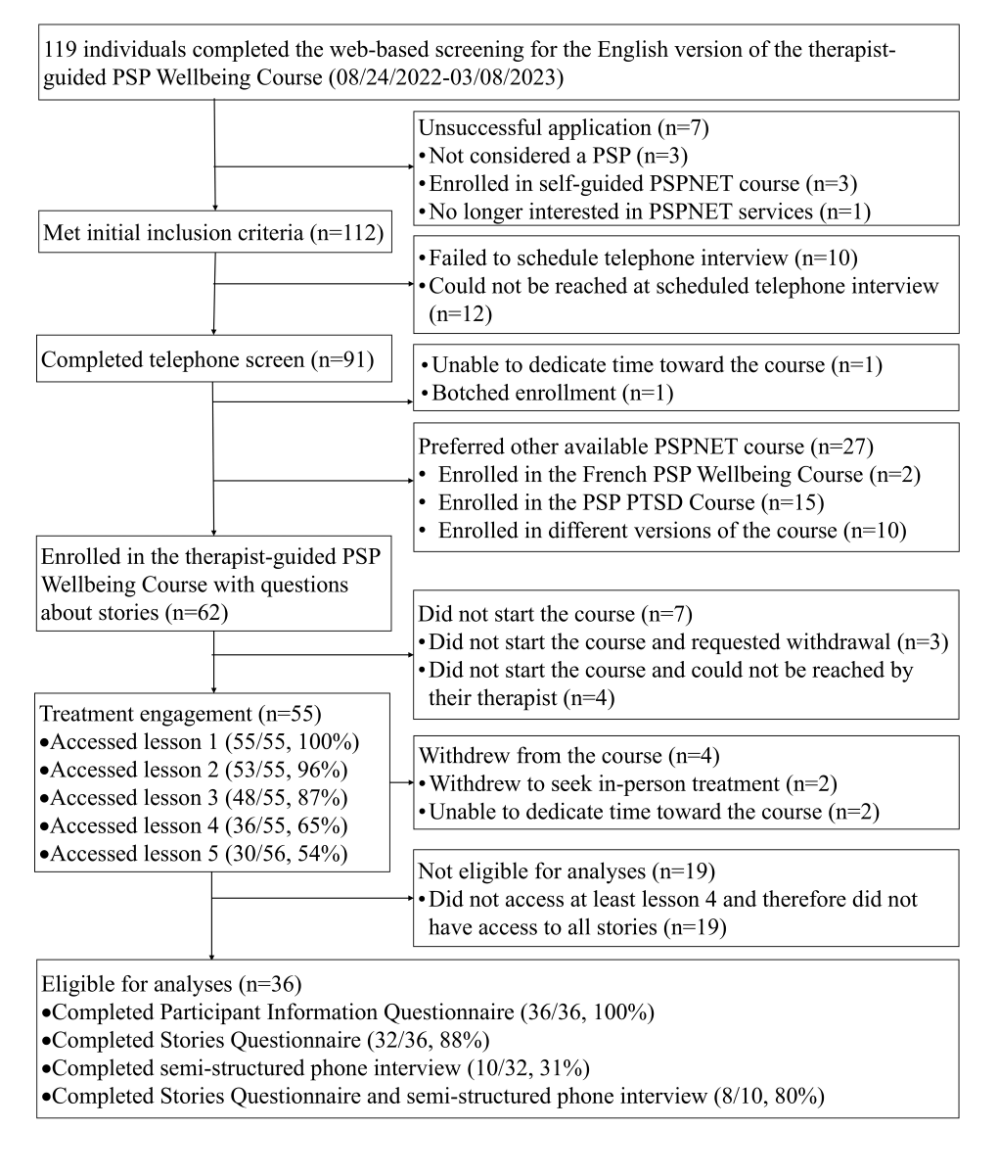
Client flowchart.

Web-based screening took place between August 2022 and March 2023 and resulted in a sample of 36 PSP (Figure 1). Eligibility criteria required that all clients who enroll in the English version of the therapist-guided PSP Wellbeing Course to (1) be 18 years or older; (2) be current or former PSP; (3) have internet access; (4) provide local emergency medical contact if presenting with severe symptoms; and (5) live in New Brunswick, Nova Scotia, Prince Edward Island, Québec, or Saskatchewan. Individuals were excluded from the PSP Wellbeing Course, and correspondingly, this study (and referred to other services as appropriate) based on the following criteria: (1) reporting high risk of suicide; (2) reporting a suicide attempt or hospitalization for high suicide risk within the past year; (3) reporting primary problems of psychosis, mania, or severe alcohol or drug use; (4) being in receipt of psychological treatment from other health care services more than twice a month; (5) not being in Canada during the treatment period; and (6) no longer being interested in service (eg, seeking other services, no longer requiring services, and being unable to devote time). Furthermore, this study only included clients who accessed at least the first 4 of 5 lessons in the English version of the PSP Wellbeing Course to ensure adequate exposure to the case stories to provide feedback.

### Measures

#### Participant Information Questionnaire

During web-based screening, prospective clients completed a Participant Information Questionnaire. This questionnaire solicited demographic data including age, gender, ethnicity, marital status, number of children, province, and education. This questionnaire also solicited occupational data (eg, PSP sector and years of experience).

#### Stories Questionnaire

The Stories Questionnaire was a bespoke questionnaire administered at 8 weeks after enrollment. It included branch logic and was composed of 4-13 items, depending on clients’ responses, assessing clients’ perceptions of and feedback on the case stories in the PSP Wellbeing Course. If clients reported reviewing at least 1 of the case stories, 9 items appeared asking for their level of agreement with the provided statements on a 5-point Likert-type scale ranging from 1 (strongly disagree) to 5 (strongly agree). Clients were asked whether the case stories represented relatable and authentic PSP experiences. Five items assessed the degree to which clients perceived the stories as fulfilling the 5 purposes of stories proposed in Shaffer and Zikmund-Fisher’s [21] taxonomy (ie, inform, comfort, model, persuade, and engage). Subsequently, clients completed 3 open-ended items inquiring about their likes, dislikes, and suggestions for story improvement. Cronbach α for Likert-type items was excellent (α=0.94). The Stories Questionnaire is shown in Multimedia Appendix 1.

#### Semistructured Interview

At 6 weeks after enrollment, a PSPNET team member invited clients to schedule a semistructured interview via email. Semistructured interviews were only conducted with the first 10 clients interested, lasted 15-60 minutes, and were scheduled for around 8 weeks after enrollment. A research assistant (JG) conducted the interviews by phone, and clients provided verbal consent to the interview and recording of the interview. Interview questions assessed clients’ perceptions of and feedback on the case stories. Clients were specifically asked questions relating to (1) perceptions of the case stories’ authenticity and relatability, (2) perceptions of the degree to which the case stories adhered to the taxonomy proposed by Shaffer and Zikmund-Fisher [21] (ie, inform, comfort, model, persuade, and engage), (3) overall perceptions of the helpfulness or unhelpfulness of the case stories, and (4) feedback for improving the case stories. Given the semistructured nature of the interview, the research assistant (JG) also asked follow-up questions where appropriate to gather more insight into story perceptions including more detailed feedback for improving the stories.

#### The PSP Wellbeing Course

The PSP Wellbeing Course is an 8-week transdiagnostic ICBT program composed of 5 lesson slideshows featuring instructive text, diagrams, and case stories that exemplify CBT coping strategies. The first lesson introduces clients to the cognitive behavioral treatment model and how to identify symptoms of mental disorders. The second lesson provides information about maladaptive thoughts and how to challenge them. The third lesson focuses on managing physical symptoms of under and overarousal. The fourth lesson teaches the implementation of graded exposure to manage behavioral symptoms. The fifth lesson concludes with information on preventing symptom relapse and sustaining treatment outcomes [27]. Throughout the course, clients also receive optional therapist support via secure email or phone up to twice a week for up to 16 weeks. Therapists provide encouragement, clarification on course material, and support to help clients integrate CBT skills into their daily lives.

Each lesson offers a supplementary video summary and downloadable PDF resources (eg, frequently asked questions, do-it-yourself guides, and additional case stories not included in the lesson slides). Additional resources cover various topics relating to mental health and situational issues (eg, sleep problems, anger, and grief) and are accessible at any time throughout the course. The lesson slideshows include text narratives of 2 fictional characters (ie, Chris [Multimedia Appendix 2] and Mike) who discuss their experiences working through the PSP Wellbeing Course and how they implemented the CBT skills outlined in each lesson. Supplementary materials include additional case stories featuring six other fictional characters who discuss their experiences, impressions of the course, successes, and challenges faced while working through course material (Table 1). Clients in the PSP Wellbeing Course are given access to all course materials for up to 1 year.

**Table 1:** Story characters.

Name (age [years] and gender)	PSP^a^ sector	Primary symptoms	Significant life events	Core thoughts	Core behaviors
Chris^b^ (40s, woman)	Police	Depression; trouble sleeping	Traumatic work events	Failure; burden to family; lazy	Social isolation; avoidance; alcohol use
Mike^b^ (50s, man)	Not specified	Anxiety; panic attacks; depression	N/A^c^	Excessive worry; self-conscious; unable to cope	Unproductive at work; avoidance
Deborah (57, woman)	Public Safety Communications	Anxiety; depression; panic attacks	Cancer survivor; children are moving out; stressful work calls	Excessive worry; family is not safe; distrust	Overeating; overprotective of children; pulling out hair
Greg (43, man)	Police	Chronic pain; depression; trouble sleeping	Motor vehicle accident at work; death of a colleague	Guilt; burden to family and colleagues	Social isolation; overuse of pain medication; alcohol and drug use
Jeff (28, man)	Corrections	Anger; trouble sleeping	Fighting with girlfriend; overwhelmed at work; surgery recovery	Unable to cope; burden to family; distrust	Aggression; alcohol use; social isolation
Lisa (55, woman)	Police	PTSD^d^	Assaulted at work	Shame; self-blame; guilt; hopelessness; burden to colleagues	Avoidance; hypervigilance; alcohol use
Mark (28, man)	Fire	Anger; panic attacks; social anxiety	Expecting first baby; training; work schedule changes	Self-doubt; poor social skills	Social isolation; angry outbursts; overuse of prescription drugs
Nicole (32, woman)	Paramedic	Depression; trouble sleeping	Caring for ill mother; overwhelmed at work	Inadequacy; letting family down; unable to care for self	Alcohol and tobacco use; unhealthy eating; social isolation

^a^PSP: public safety personnel.

^b^Story included in the core content of each lesson.

^c^Not applicable.

^d^PTSD: posttraumatic stress disorder.

### Data Analyses

Quantitative data were analyzed using SPSS (version 28; IBM Corp) to provide descriptive statistics from all eligible clients (n=36) via the Patient Information Questionnaire and Stories Questionnaire. Qualitative data were then analyzed using NVivo 20 (QSR International) analysis software [30] using a reflexive approach to open-ended responses via the Stories Questionnaire (n=25) and the semistructured interviews (n=10). All client data were deidentified prior to analyses, and semistructured interviews were transcribed using a professional transcription service. The reflexive analysis was conducted by identifying, analyzing, and reporting coherent and meaningful patterns in the data [31]. Overarching topics were categorized as main themes and evaluated for any underlying topics using deductive and inductive approaches. The initial codebook was created by JG, revised by the primary coder (JABP), and reviewed by CAL. Upon completion of coding, each theme was assessed for semantic meaning and given an appropriate name [31].

## Results

### Client Flow and Demographics

Figure 1 shows the screening, enrollment, and treatment process. The mean client age was 41.4 (SD 11.3) years. Most clients resided in Saskatchewan (n=24, 67%) and self-identified as White (n=32, 89%), women (n=17, 47%), married (n=22, 61%), and parents (n=56, 56%). Clients also self-identified most frequently as police (n=17, 47%), with others self-identifying as paramedics (n=4, 11%), firefighters (n=6=, 17%), correctional workers (n=4, 11%), or other PSP sectors (n=5, 14%; Table 2). Overall, most clients who started the PSP Wellbeing Course self-reported that they reviewed at least one of the case stories (n=25, 69%).

**Table 2:** Client demographics.

		All clients (N=36)	Completed the Stories Questionnaire (n=32)			Did not complete the Stories Questionnaire (n=4)
			Reviewed at least 1 story (n=25)	Did not review any stories (n=7)		
**Age (years), mean (SD)**		42.9 (11.1)	44.7 (10.1)	40.3 (15.5)	36.6 (4.7)	
**Gender, n (%)**						
	Woman	17 (47)	13 (52)	1 (14)	3 (75)	
	Man	19 (53)	12 (48)	6 (86)	1 (25)	
**Ethnicity, n (%)**						
	White	32 (89)	22 (88)	6 (86)	4 (100)	
	Other	4 (11)	3 (12)	1 (14)	0 (0)	
**Married or common law, n (%)**						
	Yes	22 (61)	17 (68)	3 (43)	2 (50)	
	No	14 (39)	8 (32)	4 (57)	2 (50)	
**Children, n (%)**						
	Yes	20 (56)	16 (64)	3 (43)	1 (25)	
	No	16 (44)	9 (36)	4 (57)	3 (75)	
**Home province, n (%)**						
	Maritimes	11 (31)	9 (36)	2 (29)	0 (0)	
	Saskatchewan	24 (67)	15 (60)	5 (71)	4 (100)	
	Other	1 (3)	1 (4)	0 (0)	0 (0)	
**PSP** ^a^ **sector, n (%)**						
	Corrections	4 (11)	1 (4)	2 (29)	1 (25)	
	Fire	6 (17)	3 (12)	3 (43)	0 (0)	
	Paramedics	4 (11)	3 (12)	0 (0)	1 (25)	
	Police	17 (47)	13 (52)	2 (29)	2 (50)	
	Other	5 (14)	5 (20)	0 (0)	0 (0)	
**Years in PSP field, mean (SD)**		13.8 (10.7)	14.0 (9.3)	17.4 (15.5)	5.8 (4.9)	
**Highest level of education, n (%)**						
	High school diploma or less	2 (6)	1 (4)	1 (14)	0 (0)	
	Some college or university	11 (31)	7 (28)	4 (57)	0 (0)	
	College or university degree	23 (64)	17 (68)	2 (29)	4 (100)	

^a^PSP: public safety personnel.

### Story Perceptions

Story perceptions were evaluated using quantitative data from the Stories Questionnaire for clients who self-reported viewing at least 1 of the case stories (25/36, 69%). Descriptive statistics showed of the 25 clients, most agreed or strongly agreed that “I could relate to at least one story” (n=15, 60%) and that “the stories show a bias-free perspective about what it is like to be a PSP” (n=21, 84%). Consistent with expectations, story perceptions in the PSP Wellbeing Course were generally positive with most clients endorsing the case stories as fulfilling the taxonomy proposed by Shaffer and Zikmund-Fisher [21]. Results showed that most clients agreed or strongly agreed that “I find that the stories are a trustworthy source of information” (n=16, 64%), “at least one story increased my knowledge about my mental health” (n=18, 72%), “reading the stories made me realize I am not alone with my mental health experiences” (n=18, 72%), “at least one story gave me ideas about how to use the skills to improve my well-being” (n=18, 72%), and “at least one story motivated me to use the skills” (n=16, 64%). Most clients also agreed or strongly agreed that “at least one story helped me better understand the lesson content” (n=14, 56%) and “at least one story made me want to continue on with the course” (n=14, 56%; Table 3).

**Table 3:** Story perceptions (n=25).

	Score, mean (SD)	Strongly disagree (1), n (%)	Disagree (2), n (%)	Neutral (3), n (%)	Agree (4), n (%)	Strongly agree (5), n (%)
I could relate to at least one story	3.8 (1.2)	1 (4)	3 (12)	6 (24)	5 (20)	10 (40)
I find that the stories are trustworthy source of information	3.8 (1.0)	0 (0)	3 (12)	6 (24)	8 (32)	8 (32)
The stories show a bias-free perspective about what it is like to be a PSP^a^	4.0 (0.9)	0 (0)	1 (4)	6 (24)	9 (36)	12 (36)
Reading the stories made me realize I am not alone with my mental health experience	4.2 (0.8)	0 (0)	0 (0)	7 (28)	5 (20)	13 (52)
At least one story gave me ideas about how to use the skills to improve my well-being	3.9 (0.8)	0 (0)	1 (4)	6 (24)	12 (48)	6 (24)
At least one story motivated me to use the skills	3.8 (1.1)	1 (4)	2 (8)	6 (24)	8 (32)	8 (32)
At least one story increased my knowledge about my mental health	3.8 (0.9)	0 (0)	1 (4)	9 (36)	9 (36)	6 (24)
At least one story helped me to better understand the lesson content	3.6 (1.3)	2 (8)	3 (12)	6 (24)	7 (28)	7 (28)
At least one story made me want to continue on with the course	3.5 (1.4)	3 (12)	3 (12)	5 (20)	6 (24)	8 (32)

^a^PSP: public safety personnel.

### Story Feedback

Story feedback was evaluated using qualitative data from the Stories Questionnaire and interview to gain insight on ways to improve the case stories in the PSP Wellbeing Course. Clients recommended improvements to characters, content, and delivery (Table 3). Character feedback focused on demographics and photos. Clients most frequently requested improving character demographics by incorporating more ethnicities (eg, Indigenous PSP), sectors (ie, coroner service personnel, office worker), and employment status (eg, retired PSP). Some clients also recommended using real PSP photos instead of stock photos (Table 4).

**Table 4:** Story feedback (n=27).

Themes			Example quotes		Count, n (%)
**Characters**					6 (22)
	**Demographics**			4 (15)	
		Employment status	“This is really, I...I think for a person that is still in uniform.”	1 (4)	
		Ethnicity	“More racial/cultural diversity.”	2 (7)	
		Sector	“Add the story of a PSP^a^ who works in an office, supporting field PSP and who is not a call center employee.”	2 (7)	
	**Photos**		“The pictures are obviously stock photos which adds to the fictitious feel of the story.”	2 (7)	
**Content**					5 (19)
	**Topics**			4 (15)	
		Failure	“That failure thing was overwhelming within the stories.”	1 (4)	
		Family	“I would have gotten more out of listening to the more fulsome story, like, you know, this is...this is the impact it’s had. You know, whether it’s the daughter or the son, or the husband, or the wife or somebody who...who, you know, saw their loved ones and the helpless feeling they have.”	1 (4)	
		Grief	“I came into this course with my own selfish goal. And that was this grief. If there is something here that...that could help me. And the only thing that was on here was...it was in the...of those files.”	1 (4)	
		Positivity	“We need some stories where people somehow turn that energy...that negative energy...and make it positive energy.”	1 (4)	
		PPTE^b^	“More background on some of the situations that the PSP faced prior to feeling this way, could be helpful.”	1 (4)	
		Relationships	“How to deal with relationships, uh, would probably have been really helpful.”	1 (4)	
	**Vocabulary**		“The word fear isn’t in there. And that was a word that I was using. Scared to death. You know? Like, scared and fear. And I know those words.”	2 (7)	
**Delivery**					9 (33)
	**Formatting**			2 (7)	
		Labels	“Label who the person is not just by the name but their age and occupations makes it easier for some people to know who is who.”	1 (4)	
		Navigational links	“I’d actually put a reference in there, and this is how I solved it, or this is how I dealt with it. A little click link and it’s in the material on the same module.”	1 (4)	
	**Media**		“I think it would be a different...a different reaction even if that, you know, if you had a person sitting in front of you that told you the story. Or, um, or even, like, a short video.”	1 (4)	
	**Realism**		“I didn’t get the impression that I was sitting down listening to somebody tell their story.”	6 (22)	
	**Quantity**		“Have more options. I think if there were even more stories, there would be even more things for people to relate to and learn from.”	2 (7)	

^a^PSP: public safety personnel.

^b^PPTE: potentially psychologically traumatic event.

Content feedback focused on modifying topics and vocabulary. Specifically, six topics were highlighted for improvement: (1) failure, (2) family, (3) grief, (4) positive self-talk, (5) PPTE, and (6) relationships. One client requested reducing the emphasis on failure in the case stories while other clients suggested adding more content on the impact of PSP mental health challenges on family members and how to navigate these relationships. Other topics that clients suggested for inclusion are grief, positive self-talk in the workplace, and PPTEs that precede characters’ mental health challenges. Clients further recommended tailoring story vocabulary to include terms more familiar to PSP such, as “fear” and “scared” in anxiety-related content, as well as the phrase “caring for those who care” among the general content (Table 4).

Delivery feedback focused on improving story formatting, media, realism, and quantity. Clients requested modifying story formatting by adding labels to improve recognition of characters throughout the course and inserting links to easily navigate from the case stories to relevant course material. One client advocated for the addition of video content in the case stories. Clients most frequently requested improving realism, citing a fictitious feel to the case stories (Table 4).

## Discussion

### Principal Findings

#### Overview

There is a growing body of research demonstrating the value of ICBT as an effective and convenient means of treatment for various mental disorders among the general population [6,32]. To increase access to ICBT among Canadian PSP, PSPNET developed the PSP Wellbeing Course. Findings show that the PSP Wellbeing Course can reduce symptoms of anxiety, depression, and posttraumatic stress [14,15]. Previously identified areas for improvement include the case stories [27]. This study expands on previous research by showing that PSP’s perceptions of the case stories in the PSP Wellbeing Course were generally positive and that PSP perceived the case stories as adhering to the taxonomy proposed by Shaffer and Zikmund-Fisher [21]. Nevertheless, findings identified 3 tangible areas for story improvement: characters, content, and delivery.

#### Characters

Most clients who accessed the PSP Wellbeing Course reviewed at least 1 of the case stories. This finding is consistent with previous research showing that many—but not all—clients engage with case stories embedded in internet-delivered interventions [33,34]. Story engagement begins by creating a connection between the client and the character via authentic [34] and relatable experiences [35]. This study found that most clients perceived the case stories as genuine and identifiable representations of PSP. Still, consistent with previous research [26,36], some clients questioned character demographics, citing a lack of diversity. The legitimacy of the characters was also questioned as some clients indicated that the photos appeared artificial. Improving character representation is important as it could enhance story engagement [37,38]. These findings highlight the need for and potential benefits of character development in the case stories in the PSP Wellbeing Course and other internet-delivered interventions.

Characters are typically designed to represent groups of target clientele that share common features [23]. Common features can yield high recognition among clients [39], but they can also lead to systematic errors such as gender and ethnic profiling [40]. Soliciting client feedback in development can help avoid such errors [39]. Clients in this study requested increasing character diversity with respect to employment status, ethnicity, and sector. Diversifying characters can increase representation [41], which may, in turn, lead to improved engagement. Clients in this study also requested the use of real PSP photos. Character photos support story engagement and empathy by creating a connection between photos and content [37]. The case stories in the PSP Wellbeing Course currently use stock photos. Stock photos are considered a convenient and cost-effective solution for case stories, but their use may hinder character authenticity and relatability [37,42]. In lieu, researchers recommend using consistent quality, head-and-shoulder photos [42] of real people in real situations [37,42]. While increasing character diversity may pose new challenges for selecting appropriate photos, we foresee the benefits of real photos outweighing these potential challenges. Still, real photos may not always be feasible in all contexts due to privacy concerns and regulations (eg, codes of conduct among provincial colleges of psychology in Canada) forbidding the use of client testimonials [43].

#### Content

The PSP Wellbeing Course was successful in achieving the five purposes of case stories proposed by Shaffer and Zikmund-Fisher [21]. To recall, case stories (1) provide information on basic knowledge and lived experiences, (2) provide comfort by normalizing experiences, (3) model targeted behaviors, (4) motivate the use of targeted behaviors, and (5) enhance engagement by increasing understanding and use of course material. Findings showed that clients most often endorsed the case stories as a source of comfort that modeled targeted behaviors, whereas clients least often endorsed the case stories as enhancing their understanding and engagement with the course material. These discrepancies were minimal and were not endorsed by all clients. Consistent with previous research [17], clients recommended expanding the depth and variety of topics discussed in the case stories but the nature of the topics was highly diverse with each client having a different recommendation. It would be impractical to add new stories to address every topic recommended by clients, and a topic recommended by a single past client would not necessarily be perceived as relevant or important by future clients; therefore, efforts to address a greater range of topics should likely begin with the topics that are recommended most frequently by multiple clients. Vocabulary can play a critical role in communicating about such topics [37,42]. Consistent with previous research [42], clients also recommended prioritizing vocabulary familiar to the clientele. Research suggests avoiding jargon and unexplained acronyms, which can result in increased treatment withdrawals [42]. Therefore, improving topics and vocabulary in case stories may indirectly improve client comfort, motivation, persuasion, and engagement.

#### Delivery

Clients highlighted four areas for improving story delivery: formatting, media, realism, and quantity. Formatting recommendations focused on increasing the accessibility of stories. Accessibility ensures that all current and prospective clients have the same opportunities to succeed regardless of physical or mental disabilities [44]. Accessibility can also increase acceptance and engagement in internet-delivered interventions [44]. To improve story accessibility in the PSP Wellbeing Course, clients suggested adding character labels to facilitate recognition throughout the course, and navigational links to relevant course material. To further promote accessibility, case stories could incorporate media content as not all clients support text-only narratives in case stories [17,26,36]. Benefits of audiovisual narratives include decreased cognitive load [38], as well as increased engagement [34,45] and persuasion [22,45]. Shaffer and Zikmund-Fisher [21] explain that audiovisual narratives require less cognitive resources compared to text narratives. Decreasing cognitive load means that clients have more cognitive resources available to engage in internet-delivered interventions [22]. Audiovisual narratives can also be more persuasive in changing client attitudes or behaviors compared to text narratives [22,45,46]. For example, clients report higher empathy for audiovisual characters [45]. Incorporating audiovisual narratives into case stories in the PSP Wellbeing Course may then be cost-effective when seeking to improve treatment engagement, persuasion, and accessibility.

Perceived realism refers to the degree to which characters and content resemble real people and situations [47]. Consistent with previous research [17,26], some clients recommended improving the perceived realism of case stories in the PSP Wellbeing Course. Improving perceived realism can impact clients’ identification with the characters and content [48], and in turn, client engagement [49] and persuasion of attitudes [50]. Actionable steps may include co-design via PSP working groups and adding a new survey question that asks clients to share elements of their own stories to help inform future case stories.

Overall, consistent with previous research [17], clients recommended adding more case stories to the PSP Wellbeing Course. While increasing the number of case stories may increase character representation [51], it may hinder character adherence [52]. Nevertheless, the ideal quantity of case stories in internet-delivered interventions is unclear. Casts typically vary between 3 and 12 characters [53]. The PSP Wellbeing Course maintains this norm with 8 characters. Adding more case stories may help address character diversity and other delivery issues, but such issues could also be addressed by revising existing characters and stories instead.

### Limitations and Future Directions

This study had several limitations that can further inform future research directions. First, the sample size was relatively small. Second, this study may be at risk of sampling bias, as clients with more favorable perspectives on the PSP Wellbeing Course or PSPNET may have been more receptive to PSPNET’s request for them to complete questionnaires and interviews than clients with less favorable perspectives, possibly leading to an overrepresentation of favorable perceptions of the stories in our sample. Relatedly, our exclusion of clients who had completed fewer than 4 of the 5 lessons of the course from this study may have resulted in the systematic exclusion of clients with unfavorable perspectives on the course as well. Further, most clients self-identified as White, police, or residing in Saskatchewan, and results may have limited generalizability to other groups of PSP. Third, some clients may have provided feedback without reviewing all case stories, resulting in recommendations already addressed in the case stories they did not review. Overall, results should be interpreted with caution. Future research should consider investigating whether improving the case stories in the PSP Wellbeing Course as described in this study impacts client perceptions, client engagement with treatment, and mental health outcomes. Future research could help identify perceptions of and feedback on case stories in other PSPNET courses. Finally, future research could explore whether artificial intelligence could be useful for personalizing case stories and its impact on treatment outcomes [54].

### Conclusions

This study adds to the growing body of research on the use of case stories in internet-delivered interventions. First, clients’ perceptions of the case stories in the PSP Wellbeing Course were largely positive and adhered to the taxonomy proposed by Shaffer and Zikmund-Fisher [21]. Second, client feedback provides tangible ways to improve the case stories in the PSP Wellbeing Course. Client feedback specifically identified 3 areas for story improvement: characters, content, and delivery. Each area highlights the need for and potential benefits of further story development. These findings should be interpreted with caution due to the small sample size and possible concerns with sampling and response bias. Overall, this study adds to the growing body of research supporting the use of case stories in internet-delivered interventions among PSP.

## Appendix

Multimedia Appendix 1 Stories questionnaire.

Multimedia Appendix 2 Case story sample.

## Abbreviations

CBT: cognitive behavioral therapy
ICBT: internet-based cognitive behavioral therapy
PPTE: potentially psychologically traumatic events
PSP: public safety personnel
