# Supplementary material for: Client Perspectives of Case Stories in Internet-Delivered Cognitive Behavioral Therapy for Public Safety Personnel: Mixed Methods Study
Source: JMIR Form Res. 2024 Oct 25;8:e64454. doi: 10.2196/64454 (PMC11549581; doi:10.2196/64454)
Supplement: Multimedia Appendix 1 [file formative_v8i1e64454_app1.pdf]

Stories Questionnaire

1. I reviewed some of the stories.

a. Yes
b. No

2. I could relate to at least one story.

1 2 3 4 5 0 – Did not read the stories
Strongly Disagree Strongly Agree

3. I find that the stories are a trustworthy source of information.

1 2 3 4 5 0 – Did not read the stories
Strongly Disagree Strongly Agree

4. The stories show a bias-free perspective about what it is like to be a PSP.

1 2 3 4 5 0 – Did not read the stories
Strongly Disagree Strongly Agree

5. Reading the stories made me realize I am not alone with my mental health experiences.

1 2 3 4 5 0 – Did not read the stories
Strongly Disagree Strongly Agree

6. At least one story gave me ideas about how to use the skills to improve my wellbeing.

1 2 3 4 5 0 – Did not read the stories
Strongly Disagree Strongly Agree

7. At least one story motivated me to use the skills.

1 2 3 4 5 0 – Did not read the stories
Strongly Disagree Strongly Agree

8. At least one story increased my knowledge about my mental health.

1 2 3 4 5 0 – Did not read the stories
Strongly Disagree Strongly Agree

9. At least one story helped me to better understand the lesson content.

1	2	3	4	5	0 – Did not read the stories
Strongly Disagree				Strongly Agree	

10. At least one story made me want to continue on with the course.

1	2	3	4	5	0 – Did not read the stories
Strongly Disagree				Strongly Agree	

11. Overall, what did you like about the stories?

12. Overall, what did you dislike about the stories?

13. Do you have any suggestions to help us improve the stories?
