# Supplementary material for: Client Perspectives of Case Stories in Internet-Delivered Cognitive Behavioral Therapy for Public Safety Personnel: Mixed Methods Study
Source: JMIR Form Res. 2024 Oct 25;8:e64454. doi: 10.2196/64454 (PMC11549581; doi:10.2196/64454)
Supplement: Multimedia Appendix 2 [file formative_v8i1e64454_app2.pdf]

Appendix X: Sample Case Story Excerpt

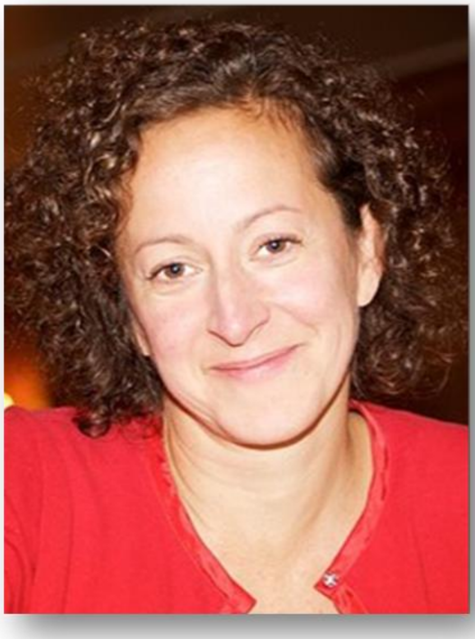

Hi, I'm Chris.

I hope my story helps.

I work in public safety, I'm in my early 40s, and have a great family (amazing spouse and 2 great kids).

Looking back, I can see that I have had three episodes of depression. The first two were mild, but the last one was really tough and I can now see how it was made worse by things that happened at work.

I had been working very hard – doing extra shifts because we were short-staffed, and it was the holiday season, so there were a lot of horrific accidents. I don't think it was any one thing that triggered it. But over a two month period I started to feel really tired, sad, and hopeless.

I noticed that the difficult calls were becoming harder for me to do – like attending a motor vehicle incident with fatalities. I was still competent at my job, but I felt like I was losing my passion. I began to get irritable, snapping for no reason. And then I'd feel immense guilt.

My mood got really dark at times, and I started worrying about my family and also about most other things. Even little things started to upset me and I started to feel more tense and jumpy, and wasn't sleeping properly.

I stopped going out, and even taking the kids to the park became a chore. Then I started to feel even more guilty.

It was hard to admit, but I was starting to think life was meaningless …

… I wasn't suicidal, but I just felt like I didn't care anymore. Finally, I talked to my partner at work who had been worried about me for a while. I broke down. It was a relief to be honest and I agreed to see my family doctor and talk to my supervisor.

My supervisor actually suggested I try an online treatment program … I didn't know what to expect. But from the first lesson I felt confident it was worth trying. I'll share my story throughout the Course.

Good luck!

- Chris
